# Stent-free biliary bypass by endoscopic ultrasound-guided choledochoduodenostomy for benign common hepatic duct stricture

**DOI:** 10.1055/a-2837-1328

**Published:** 2026-04-20

**Authors:** Yuki Uba, Kazuo Hara, Nozomi Okuno, Shin Haba, Takamichi Kuwahara, Shimpei Matsumoto, Hiroki Koda

**Affiliations:** 1 538363 Department of Gastroenterology, Aichi Cancer Center Hospital, Nagoya, Japan

Benign biliary strictures are usually managed by transpapillary stenting with periodic exchanges 1 . For strictures just below the hepatic confluence, long-term stent dependence is a limitation 2 3 . We present a two-stage choledochoduodenostomy creating a proximal common hepatic duct bypass anastomosis cranial to the stricture, aiming for stent-free patency ( Video 1 ).

Two-stage choledochoduodenostomy for benign common hepatic duct strictures near the hilum. A plastic stent matured the anastomosis, followed by bilateral slim fully covered metal stents, enabling stent-free patency.Video 1

**Fig. 1:**
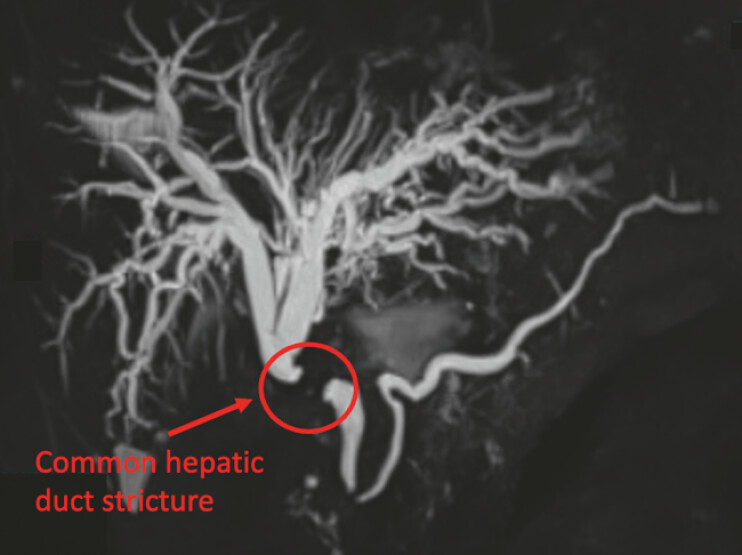
MRCP showing a short-segment common hepatic duct stricture immediately below the hepatic confluence. MRCP, magnetic resonance cholangiopancreatography.

Elsewhere, a man in his 40s underwent endoscopic retrograde cholangiopancreatography (ERCP) for choledocholithiasis, complicated by post-ERCP pancreatitis. After recovery, liver dysfunction was noted; magnetic resonance cholangiopancreatography revealed a common hepatic duct (CHD) stricture just below the hilum, likely due to inflammatory spread ( Fig. 1 ). ERCP management was considered, but repeated stent exchanges were expected; therefore, we chose endoscopic ultrasound-guided choledochoduodenostomy to bypass the obstruction and pursue stent-free patency.

Stage 1: From the duodenal bulb, the CHD upstream to the stricture was punctured with a 22-gauge needle. A 0.018-inch guidewire was advanced into the intrahepatic bile ducts, and the tract was dilated with a drill dilator without electrocautery. A 7-Fr × 7-cm plastic stent was placed into the right hepatic duct via choledochoduodenostomy to allow anastomosis maturation. No adverse events occurred, and the patient was discharged 7 days later.

Stage 2 (2 months late): Cholangiography through the anastomosis demonstrated the CHD stricture caudal to the anastomosis, consistent with a bypass. After re-dilation with a 4-mm balloon, two slim 6-mm × 6-cm fully covered metal stents were deployed toward the right and left hepatic ducts to secure bilateral drainage.

**Fig. 2:**
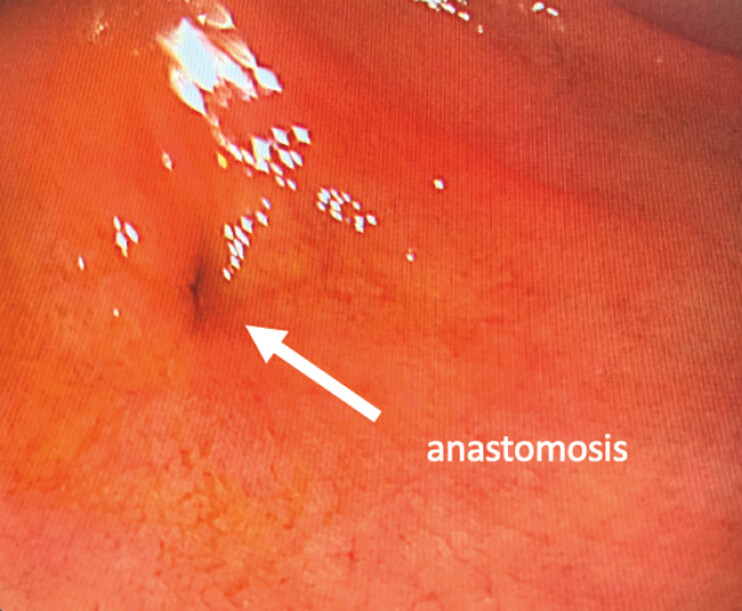
Upper endoscopy at 18 months confirming a patent choledochoduodenostomy anastomosis.

Two months later, all stents were removed. Cholangiography confirmed a patent anastomosis with unobstructed bilateral bile flow, and hepatobiliary scintigraphy 1 month later showed adequate excretion. No procedure-related complications occurred. At 18 months, upper endoscopy confirmed a patent choledochoduodenostomy anastomosis ( Fig. 2 ), and the patient has remained stent-free without cholangitis.

Endoscopy_UCTN_Code_TTT_1AR_2AL
